# Role of Extended Mesenteric Excision in Postoperative Recurrence of Crohn's Colitis: A Single-Center Study

**DOI:** 10.14309/ctg.0000000000000407

**Published:** 2021-10-01

**Authors:** Yipeng Zhu, Wenwei Qian, Liangyu Huang, Yihan Xu, Zhen Guo, Lei Cao, Jianfeng Gong, J. Calvin Coffey, Bo Shen, Yi Li, Weiming Zhu

**Affiliations:** 1 Department of General Surgery, Jinling Hospital, Medical School of Southeast University, Nanjing, PR China;; 2 Department of General Surgery, Jinling Hospital, Medical School of Nanjing University, Nanjing, PR China;; 3 Department of General Surgery, Jinling Hospital, School of Nanjing Medical University, Nanjing, PR China;; 4 Department of Surgery, University Hospital Limerick, Limerick, Ireland;; 5 Section of Inflammatory Bowel Diseases and Center for Interventional IBD, Columbia University Irving Medical Center-New York Presbyterian, New York, New York, USA.

## Abstract

The mesentery is involved in Crohn's disease. The impact of the extent of mesenteric resection on postoperative disease progression in Crohn's disease remains unconfirmed. This study aimed to determine the association between resection of the mesentery and postoperative outcomes in patients with Crohn's colitis (CC) undergoing colorectal surgery.

**METHODS:**

Patients with CC who underwent colorectal resection between January 2000 and December 2018 were reviewed, and the data were gathered from a prospectively maintained database. Patients were divided into 2 groups according to the extent of mesenteric resection, the extensive mesenteric excision (EME) group and the limited mesenteric excision (LME) group. Outcomes including early postoperative morbidities and surgical recurrence were compared between the 2 groups.

**RESULTS:**

Of the 126 patients included, 60 were in the LME group and 66 in the EME group. There was no significant difference between the 2 groups in early postsurgical outcomes except the intraoperative blood loss was increased in the LME group (*P =* 0.002). Patients in the EME group had a longer postoperative surgical recurrence-free survival time when compared with those in the LME group (*P =* 0.01). LME was an independent predictor of postoperative surgical recurrence (hazard ratio 2.67, 95% confidence interval 1.04–6.85, *P =* 0.04). This was further confirmed in the subgroup analysis of patients undergoing colorectal resection and anastomosis (hazard ratio 2.83, 95% confidence interval 1.01–7.96, *P =* 0.048).

**DISCUSSION:**

In patients undergoing surgery for CC, inclusion of the mesentery is associated with similar short-term outcomes and improved long-term outcomes compared with those seen when the mesentery is retained.

## INTRODUCTION

Crohn's disease (CD) is a chronic inflammatory bowel disease that can affect the entire gastrointestinal tract. Up to 78% of patients with CD will require surgical intervention (1), and approximately 20% of patients will require further surgery for disease recurrence within 10 years (2). Factors including smoking, previous intestinal surgery, absence of prophylactic treatment, penetrating disease at index surgery, and increased enteric glial cells in proximal margin are predictors of postoperative recurrence (3–8). Postoperative medical prophylaxis is associated with reduced recurrence after surgical resection (3,4,9).

Increasing data suggest that the mesentery is involved in CD (10–12). Mesenteric adipose tissue and alterations in mesenteric lymphatics correlate with disease progression of CD. Accumulation of visceral adipose tissue (13,14), increased mesenteric lymphatic-vessel density in the mesenteric resection margin (15), and the presence of granulomata in the mesenteric lymph nodes are associated with increased rates of postoperative recurrence (16). Bacterial translocation is common in mesenteric fat and may contribute to inflammatory response in CD (17). Collectively, emerging findings indicate the mesentery could represent a therapeutic target in CD.

In colorectal surgery for benign disease such as CD and ulcerative colitis, both the conservative approach (i.e., retention of the mesentery) and wide mesenteric excision (similar to complete mesocolic excision) are performed according to the individual surgeon's discretion. Given clarification of the structure of mesentery (18–21) and its potential role in the disease course of CD, the extent of mesenteric resection in surgery for CD may impact on postoperative long-term outcomes. Indeed, a recent study reported that inclusion of the mesentery in ileocolic resection for CD is associated with reduced surgical recurrence (22). In addition, a recent case report described the development of cancer postoperatively in a patient who underwent a mesentery sparing right hemicolectomy for CD (23). Given the above, it is important to further investigate the effects of mesentery removal in patients undergoing surgery for Crohn's colitis (CC). In line with this, the aim of this study was to investigate and compare the effects of extensive mesenteric excision (EME) and limited mesenteric excision (LME), on postoperative outcomes in patients with CC and who were undergoing colorectal resection.

## MATERIALS AND METHODS

### Patients

Patients with CC who underwent colorectal resection at the Inflammatory Bowel Disease Center of Jinling Hospital between January 2000 and December 2018 were reviewed.

### Inclusion and exclusion criteria

All patients undergoing colorectal resection for CC were included. Exclusion criteria were as follows: (i) age < 18 years, (ii) history of colorectal resection, (iii) concomitant jejunal or ileal resection, (iv) abdominoperineal resections for perianal disease, (v) fecal diversion without colorectal resection, or (vi) surgery for dysplasia and colorectal cancer complicating CD. Ethical approval was obtained from the Ethics Committee of Jinling Hospital.

### Surgical approach

Patients were categorized into EME and LME groups according to the extent of mesenteric resection. In colorectal surgery for CC, the mesentery mobilized with peritonoming provides access to the mesofascial plane. Mesofascial separation (i.e., the separation of components of the mesofascial plane) enabled detachment of the intact mesentery both from medial to lateral or from lateral to medial. Disconnection of the mesentery was completed when vascular mesenteric pedicles were divided. It is important to realize the root region of the mesentery is not dissected Crohn's patients because it is too dangerous in most cases. Surgery was performed by 1 group of surgeons with extensive experience in surgery for CD. LME was defined as patients undergoing mesentery sparing colorectal resection where the mesentery was divided close to the bowel wall and the mesentery thus preserved. In the EME group, the mesentery was fully mobilized and divided 1-cm distant from the origin of the major arterial trunks (i.e., similar to the oncological approach to colorectal resection). The proximal and distal resection margins were positioned at levels where the intestine was macroscopically normal. The division of mesentery, EME, or LME was performed according to individual surgeon's discretion, and the surgical approach was established from operative records. The anastomotic configuration was also collected.

### Postoperative management

All patients received the same postoperative management based on local standard practice and guidelines. To reduce postoperative recurrence, adjuvant medical treatment was given to patients with at least 1 risk factor for recurrence (24,25). Postoperative reviews were conducted at 6-monthly intervals. Patients were followed regularly to March 31, 2020, or to the development of postoperative surgical recurrence. A complete clinical, endoscopic, and radiological work-up was only instituted if clinically necessitated (i.e., if there was a suspicion of impending surgical recurrence).

### Data collection

Patient information was collected from a prospectively maintained inflammatory bowel disease database. The following data were collected: sex, age of onset, age at the index surgery, duration of disease, body mass index (BMI), Montreal classification, medications before and after surgery, history of surgery, smoking history, American Society of Anesthesiologists score, site of colorectal surgery, stoma creation, preoperative levels of C-reactive protein and serum albumin, and information of postoperative outcomes. Preoperative medication was defined as the use of medications (corticosteroids, immunosuppressants, and anti–tumor necrosis factor biologics) 1 month preoperatively. In addition, histopathological assessment of resected specimens was collated (see Supplementary Figure and Supplementary Result, Supplementary Digital Contents 1 and 2, http://links.lww.com/CTG/A692 and http://links.lww.com/CTG/A693).

### Postoperative outcomes

Early postoperative short-term outcome and surgical recurrence were evaluated. The main endpoint of the study (postoperative surgical recurrence) was defined as the requirement of reoperation for recurrent CD. Reoperations as part of management of a postoperative complication were not considered a postoperative surgical recurrence. For patients undergoing colorectal resection and primary anastomosis, the date of initial bowel resection was considered as the entry point of this study; for those having colorectal resection and stoma creation, the time of restoration of stoma was considered as the starting point of postoperative recurrence surveillance; and for those undergoing colorectal resection and stoma creation without subsequent stoma closure, the date of initial bowel resection was considered the starting point of this study. Early postoperative outcomes included postoperative morbidities, time to bowel movement, postoperative blood transfusion requirement, duration of postoperative hospitalization, readmission, and reoperation because of postoperative complications. All complications were defined as those occurring within 30 days from the date of surgery. Early postoperative morbidities were divided into infectious complications including wound infection, intra-abdominal abscess, anastomotic leak, and noninfectious complications such as postoperative ileus, intra-abdominal bleeding, and dysfunction of gastrointestinal recovery.

### Statistical analysis

Statistical analysis was performed using SPSS version 25.0 (IBM, Chicago, IL). Continuous variables are presented as the mean ± SD and were compared using the Student *t* test for normally distributed variables. The Mann-Whitney *U* test was used to analyze nonparametric data. The Fisher exact test or the χ^2^ test was used to compare categorical variables. The relative risks and 95% confidence intervals (CIs) were also calculated to identify potential effects. Survival was estimated by the Kaplan-Meier method, and any differences in survival were evaluated with a stratified log-rank test. Multivariable analysis with Cox proportional hazards model was used to estimate the simultaneous effects of factors on surgical recurrence. In addition, confounders were included in multivariable analysis to control the confounding bias. R (version 3.6.3) with RStudio (version 1.2.5033) was used to perform survival analysis and prepare figures. A *P* value of < 0.05 was considered statistically significant.

## RESULTS

### Baseline characteristics of patients

One hundred thirty-nine patients were initially eligible for inclusion in the study. Thirteen patients were excluded because of incomplete data. We included 126 patients (89 men, 70.6%) undergoing colorectal resection for CC in the final study cohort. There were 60 patients in the LME group and 66 patients in the EME group. Demographic and clinical characteristics at baseline are listed in Table 1. The mean age at surgery was 30.77 ± 10.21 years, and the mean preoperative duration of disease was 56.64 ± 50.93 months. The mean BMI of patients in the cohort was 18.20 ± 2.76 kg/m^2^. Fourteen patients (11.1%) had a smoking history, and 7 patients (5.6%) had history of previous surgery for CD. The mean blood loss was 102.18 ± 72.77 mL in the LME group and was significantly higher than in the EME group (*P =* 0.002). Age of disease onset, age at the index surgery, sex, duration of disease, BMI, preoperative medications, smoking history, American Society of Anesthesiologists score, preoperative C-reactive protein, preoperative serum albumin levels, site of colorectal surgery, stoma creation, and the disease location were all comparable between the 2 groups.

**Table 1.** Demographic data and disease characteristics of patients

	Group LME (n = 60)	Group EME (n = 66)	*P* value
Male sex, n (%)	47 (78.3)	42 (63.6)	0.07
Age of onset, mean ± SD, yr	26.85 ± 10.82	25.23 ± 9.71	0.38
Age at the index surgery, mean ± SD, yr	31.15 ± 10.36	30.42 ± 10.15	0.69
Duration of disease, mean ± SD, mo	51.19 ± 49.36	61.61 ± 52.19	0.25
BMI, mean ± SD, kg/m^2^	18.48 ± 3.19	18.04 ± 2.49	0.48
Surgery history, n (%)	6 (10.0)	1 (1.5)	0.09
Smoking history, n (%)	9 (15.0)	5 (7.6)	0.19
Preoperative medication, n (%)	32 (53.3)	38 (57.6)	0.63
Immunosuppressants	19 (31.7)	23 (34.8)	0.71
Infliximab	4 (7.1)	4 (6.3)	1.00
Corticosteroids	21 (35.0)	25 (37.9)	0.74
Age at diagnosis, n (%), yr			0.51
A1 ≤16	7 (11.7)	4 (6.1)	0.27
A2 17-40	44 (73.3)	50 (75.8)	0.76
A3 > 40	12 (15.0)	12 (18.2)	0.80
Disease location, n (%)			0.32
L2 (colonic)	32 (53.3)	41 (62.1)	0.32
L3 (ileocolonic)	28 (46.7)	25 (37.9)	0.32
L4 (upper tract)	1 (1.7)	4 (6.1)	0.42
Disease behavior, n (%)			**0.002**
B1	0 (0)	3 (4.5)	0.28
B2	20 (33.3)	39 (59.1)	**0.004**
B3	40 (66.7)	24 (36.4)	**0.001**
P	25 (41.7)	22 (33.3)	0.33
ASA score, n (%)			0.47
≥3	10 (16.7)	8 (12.1)	
<3	50 (83.3)	58 (87.9)	
Preoperative CRP, mean ± SD, mg/L	16.10 ± 27.77	12.60 ± 23.12	0.30
Preoperative albumin, mean ± SD, g/L	37.11 ± 4.99	37.28 ± 5.91	0.50
Postoperative medicine prophylaxis, n (%)	30 (50.0)	40 (60.6)	0.23
Immunosuppressants	29 (48.3)	38 (57.6)	0.30
Biologics	2 (3.3)	4 (6.1)	0.77
No immunosuppressants or biologics, n (%)	30 (50.0)	26 (39.4)	0.23
Site of colorectal surgery, n (%)			0.22
Right hemicolectomy	26 (43.3)	32 (48.5)	0.56
Transverse colectomy	4 (6.7)	3 (4.5)	0.90
Left hemicolectomy	21 (35.0)	14 (21.2)	0.08
Proctectomy	2 (3.3)	1 (1.5)	0.93
Total colectomy	7 (11.7)	16 (24.2)	0.07
Stoma creation, n (%)	14 (23.3)	11 (16.7)	0.35
Terminal ileum	8 (57.1)	9 (81.8)	0.38
Ascending colon	0 (0)	1 (9.1)	0.44
Transverse colon	3 (21.4)	0 (0)	0.31
Descending colon	3 (21.4)	1 (9.1)	0.78
Microscopic resection margin positive, n (%)			
Proximal margin	4 (8.7)	4 (7.3)	1.00
Distal margin	3 (6.5)	4 (7.3)	1.00
Stoma margin	1 (7.1)	1 (9.1)	1.00

ASA, American Society of Anesthesiologists; BMI, body mass index (calculated as weight in kilograms divided by height in m^2^); CRP, C-reactive protein; EME, extensive mesenteric excision; LME, limited mesenteric excision.

In the overall cohort, 101 (80.2%) patients underwent colorectal resection and anastomosis. Forty-six (45.5%) underwent resection and anastomosis in the LME group and 55 (54.5%) in the EME group. This meant 25 (19.8%) patients of the overall cohort underwent colorectal resection with stoma creation, 14 (56%) in the LME group and 11 (44%) in the EME group. On histopathological assessment, inflammation was apparent in 8.7% and 7.3% (*P =* 1.00) of the LME and EME groups, respectively. Histological inflammation was apparent in the distal resection margin in 6.5% and 7.3% (*P =* 1.00) of the LME and EME groups, respectively. In the cohort of patients who underwent stoma formation, histological inflammation was apparent in 7.1% and 9.1% of the LME and EME groups (*P =* 1.00), respectively. In patients who underwent anastomosis, a stapled side-to-side anastomosis was fashioned in all.

### Early postoperative short-term outcomes

There was no postoperative mortality. Thirty-one (24.6%) patients developed postoperative complications. There were 16 (51.6%) infectious complications and 15 (48.4%) noninfectious complications. Table 2 shows that 14 (23.3%) in the LME group compared with 17 (25.8%) in the EME group (*P =* 0.75) experienced a short-term postoperative complication. Postoperative hospital stay, time to return to bowel movements, blood transfusion requirement, reoperation, and readmission rates were similar between both groups.

**Table 2.** Postoperative short-term outcomes

	Group LME (n = 60)	Group EME (n = 66)	*P* value
Blood loss, mean ± SD, mL	132.25 ± 85.53	83.10 ± 56.19	**0.002**
Length of hospital stay, mean ± SD, d	12.64 ± 8.57	10.49 ± 5.17	0.47
Blood transfusion, n (%)	4 (6.7)	6 (9.1)	0.86
Time to return to bowel movements, mean ± SD, d	8.70 ± 3.71	9.13 ± 3.93	0.58
Readmission, n (%)	2 (3.3)	2 (3.0)	1.00
Reoperation, n (%)	3 (5.0)	2 (3.0)	0.91
Postoperative morbidity, n (%)	14 (23.3)	17 (25.8)	0.75
Postoperative complications, n (%)			
Wound infection	4 (28.6)	3 (17.6)	0.67
Intra-abdominal abscess	0 (0)	2 (11.8)	0.49
Anastomotic leak	5 (35.7)	2 (11.8)	0.20
Ileus	0 (0)	2 (11.8)	0.49
Intra-abdominal bleeding	2 (14.3)	0 (0)	0.20
Dysfunction of gastrointestinal recovery	2 (14.3)	6 (35.3)	0.24
Other	1 (7.1)	2 (11.8)	1.00

EME, extensive mesenteric excision; LEM, limited mesenteric excision.

### Postoperative surgical recurrence

**Figure 1.**
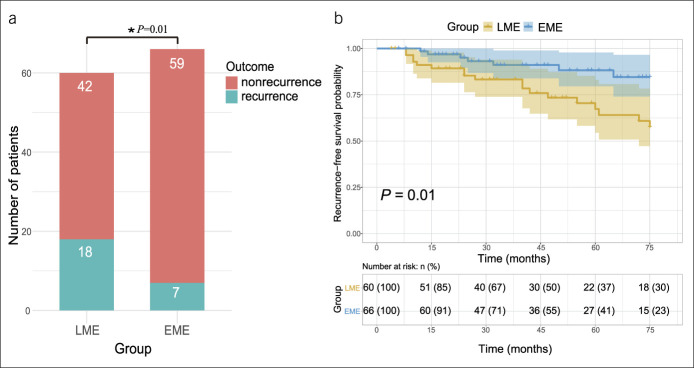
(**a**) Surgical recurrence in group LME and group EME. (**b**) Kaplan-Meier estimates demonstrating the cumulative incidence of surgical recurrence in group LME and group EME. EME, extensive mesenteric excision; LME, limited mesenteric excision. *P< 0.05.

We compared the rates of surgical recurrence between the 2 groups. The mean duration of follow-up was 45.12 ± 25.45 months and 47.50 ± 23.67 months in the LME and EME groups, respectively. In the LME group, 18 (30.0%) patients required reoperation because of recurrent CD. The mean time to reoperation was 34.94 ± 22.89 months. Seven (10.6%) patients developed postoperative surgical recurrence in the EME group. The mean time to reoperation was 31.57 ± 19.43 months. Cumulative reoperation rates were 30.0% and 10.6% in the LME and EME groups (*P =* 0.01), respectively (Figure 1a). Kaplan-Meier survival curves and the log-rank test of 2 groups were shown in Figure 1b. The data also indicate that patients in the EME group had a longer postoperative surgical recurrence-free survival time when compared with those in the LME group (*P =* 0.01).

### Risk factors for postoperative surgical recurrence

Factors known or reported to increase the risk of surgical recurrence were evaluated in univariate analysis. These included sex, age at the index surgery, smoking history, disease duration, age at diagnosis, history of surgery, postoperative medicine prophylaxis, site of colorectal resection, disease behavior, LME, and stoma creation. Next, factors with a *P* value less than 0.05 on univariate analysis (i.e., smoking history and LME) were included in a multivariate analysis (Cox regression). Disease phenotype was also included in the multivariate analysis to control for potential confounding bias. The comparison is summarized in Table 3. LME was an independent predictor of postoperative surgical recurrence (hazard ratio [HR] 2.67, 95% CI 1.04–6.85, *P =* 0.04) (i.e., recurrence requiring surgical intervention). Smoking history (HR 4.04, 95% CI 1.64–9.97, *P =* 0.002) was also an independent risk factor for postoperative surgical recurrence.

**Table 3.** Univariate and multivariate analysis of risk factors for surgical recurrence

	Univariate analysis		Multivariate analysis	
	HR (95% CI)	*P* value	HR (95% CI)	*P* value
Male sex	0.49 (0.17–1.42)	0.19		
Duration of disease	0.10 (0.99–1.01)	0.69		
Age at the index surgery	1.01 (0.97–1.05)	0.56		
Surgery history	0.94 (0.13–6.92)	0.95		
Age at diagnosis	1.02 (0.98–1.05)	0.44		
Site of resection	—	0.55		
Penetrating vs stricturing phenotype	1.47 (0.64–3.35)	0.36	1.47 (0.61–3.54)	0.40
Perianal disease	1.02 (0.46–2.26)	0.97		
Smoking history (yes vs no)	**4.00** (**1.72–9.30)**	0.001*	**4.04** (**1.64–9.97)**	0.002
Postoperative biologics vs immunosuppressants	0.04 (0.00–154.61)	0.45		
Postoperative biologics vs none	0.04 (0.00–74.64)	0.40		
Postoperative immunosuppressants vs none	0.75 (0.33–1.72)	0.50		
LME vs EME	**2.97** (**1.24–7.11)**	0.02*	**2.67** (**1.04–6.85)**	0.04*
Stoma vs primary anastomosis	0.65 (0.24–1.74)	0.39		

CI, confidence interval; EME, extensive mesenteric excision; HR, hazard ratio; LME, limited mesenteric excision.

* *P*< 0.05.

### Subgroup analysis of patients with anastomosis

**Figure 2.**
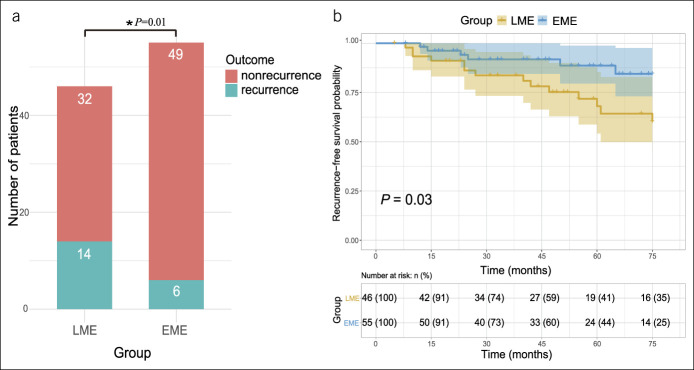
(**a**) Surgical recurrence in subgroup anastomosis. (**b**) Kaplan-Meier estimates demonstrating the cumulative incidence of surgical recurrence in group LME and group EME for subgroup anastomosis. EME, extensive mesenteric excision; LME, limited mesenteric excision. *P< 0.05.

The following is a subgroup analysis of outcomes for patients who underwent a resection and anastomosis (101, 80.2%). In patients who underwent a limited mesenteric resection and anastomosis (i.e., in the LME group [n = 46]), 14 (37.0%) required reoperation because of recurrent CD. The mean time to reoperation was 35.57 ± 21.84 months. In patients who underwent a mesenteric resection and anastomosis (n = 55), 6 (10.9%) patients experienced postoperative surgical recurrence. In this cohort, the mean time to reoperation was 31.50 ± 21.29 months. The cumulative reoperation rates were 30.4% and 10.9% in the 2 groups (*P =* 0.01), respectively (Figure 2a). Kaplan-Meier survival curves and the log-rank test of the 2 groups (Figure 2b) indicated that patients in the EME group had a longer postoperative surgical recurrence-free survival time compared with those in the LME group (*P =* 0.03). Factors associated with postoperative surgical recurrence were identified by univariate and multivariate analysis in this subgroup of patients (Table 4). On a Cox regression analysis, LME was an independent risk factor of postoperative surgical recurrence (HR 2.83, 95% CI 1.01–7.96, *P =* 0.048). Smoking history (HR 2.88, 95% CI 1.07–7.70, *P =* 0.04) was also an independent risk factor for postoperative surgical recurrence.

**Table 4.** Univariate and multivariate analysis of patients with anastomosis

	Univariate analysis		Multivariate analysis	
	HR (95% CI)	*P* value	HR (95% CI)	*P* value
Male sex	1.73 (0.71–4.23)	0.23		
Duration of disease	0.99 (0.98–1.00)	0.11		
Age at the index surgery	1.01 (0.97–1.05)	0.60		
Surgery history	0.05 (0.00–256.57)	0.49		
Age at diagnosis	1.02 (0.99–1.06)	0.21		
Site of resection	—	0.38		
Penetrating vs stricturing phenotype	1.89 (0.86–4.16)	0.11	1.22 (0.47–3.20)	0.68
Perianal disease	0.91 (0.43–1.96)	0.82		
Smoking history (yes vs no)	**2.67** (**1.18–6.01)**	**0.02** ^*^	**2.88** (**1.07–7.70)**	0.04*
Postoperative biologics vs immunosuppressants	0.04 (0.00–116.84)	0.44		
Postoperative biologics vs none	0.04 (0.00–57.02)	0.38		
Postoperative immunosuppressants vs none	0.83 (0.39–1.77)	0.63		
LME vs EME	**2.78** (**1.07–7.24)**	0.04*	**2.83** (**1.01–7.96)**	0.048*

CI, confidence interval; EME, extensive mesenteric excision; HR, hazard ratio; LME, limited mesenteric excision.

* *P*< 0.05.

## DISCUSSION

This study investigated the effects of inclusion of the mesentery on postoperative outcomes in patients undergoing colorectal surgery for CC. The data suggest that mesenteric resection was associated with reduced postoperative surgical recurrence when compared with rates of surgical recurrence when the mesentery is retained. Both smoking and retention of the mesentery were independently predictive of an increased requirement for reoperative surgery for a CD-related indication (4,26–28).

Previous studies of CD mainly focused on inflammatory processes in the intestinal mucosa and submucosa. More recently, there has been an increasing focus on the mesentery and the possibility this may have a pathobiological role in CD (19,29). Mesenteric abnormalities in CD include fat hypertrophy, extension of mesenteric fat over the surface of the associated intestine, and mesenteric (30–33). Emerging evidence suggests that mesenteric components, including nerves, blood vessels, lymphatics, fat, and connective tissues, are also implicated in the pathogenesis and progression of CD (12,34). Emerging findings suggest that mesenchymal abnormalities in the mesentery extend from this directly into the adjoining intestine (35). If these suggestions are borne out, they support the hypothesis that CD is a primary mesenteropathy (29) or at least bidirectional in terms of how it progresses at a tissue-based level (12). Therefore, it is reasonable to propose that resection of the inflamed mesenteric tissue during surgery for CD may provide improved postoperative outcomes. Indeed, Coffey et al. (22) found that inclusion of the mesentery in ileocolic resection for CD is associated with reduced surgical recurrence. In addition, retention of mesentery is associated with increased complications after proctectomy in CD (36). This association is believed to relate to persistent inflammation in the mesorectal remnant. In addition, exclusion of the mesentery at the anastomosis after resection (i.e., the Kono-S procedure) seems to be associated with reduced levels of postoperative surgical recurrence. A new antimesenteric, functional, end-to-end, handsewn ileocolic anastomosis (Kono-S) was found to be effective in preventing surgical recurrence at anastomosis (37). And, this was confirmed in a randomized clinical trial, indicating a significant reduction in postoperative endoscopic and clinical recurrence rate for patients who underwent Kono-S anastomosis (38). Although the mesentery is preserved in Kono-S anastomosis, this antimesenteric anastomosis keeps the anastomosis away from the site of anastomosis, implying the adverse effects of mesentery in postoperative recurrence. Therefore, the proinflammatory profile of mesentery in CD is demonstrated by antimesenteric Kono-S anastomosis and our wide mesenteric resection procedure because both techniques are trying to isolate the anastomosis as much as possible from the diseased mesentery (39).

The potential benefits associated with inclusion of the mesentery as part of the surgical management of CD may be explained by the fact that a proinflammatory trigger contained in the adipose compartment of the mesentery is being removed (12,40–42). As a result of this, complication rates are reduced and surgical recurrence is at the least delayed (13,14,43). Mesenteric lymphatic alterations (including increased mesenteric lymphatic-vessel density) have also been associated with postoperative disease recurrence (15,44). Furthermore, mesenteric lymph nodes represent sites to which intestinal bacteria translocate to incite and progress immunological responses (45). Bacterial translocation to mesenteric adipocytes and lymph nodes is common in patients with CD (45). Translocation has been linked to increased production of proinflammatory cytokines and sustained inflammation. In keeping with this, removal of the mesentery could attenuate these events and thus reduce local immunological responses. These findings may explain our finding, at least in part, that preserving the mesenteric tissue in surgical resection for CD could lead to adverse outcome in postoperative disease progression.

To exclude the potential effects of stoma creation on postoperative recurrence, a subgroup analysis was performed. Then results again support the suggestion that LME is a predictor of postoperative surgical recurrence. Interestingly, in a subgroup of patients undergoing colorectal resection with stoma formation, the adverse effects of LME were not apparent. The observed trend (when taken in conjunction with observations related to the Kono-S procedure) suggests that the combination of fecal flow and retention of the mesentery together provide a tissue-level environment in which recurrent disease emerges early and progresses to a level requiring surgical intervention. Further studies with a large number of patients are warranted to clarify this issue.

The question arises as to why postoperative surgical recurrence is also reduced after stricturoplasty, that is, where the mesentery is not resected nor excluded from the anastomosis. Stricturoplasty is associated with a marked change in the conformation of the intestine (the circumference of the intestine is increased). Given these, it is feasible the interaction between the intestine an adjoining mesentery is beneficially altered. Against this, however, rates of surgical recurrence after stricturoplasty vary considerably (46–52).

In addition, current data suggest that the genetics, microbiota, serology, and smoking association of isolated colonic CD lie between those of ileo/ileocolonic CD and ulcerative colitis and make a strong case for this phenotype being considered separately (53–59). Further analysis revealed significant differences in mesenteric fat cell size, fat tissue inflammation, T-cell infiltration, and fibrosis between small bowel CD and CC (60). Therefore, the study on the effect of specific mesocolon on postoperative outcomes is also conducive to further understanding the fact that CC might be phenotypically different from small bowel disease.

This study has a number of limitations. It was historical cohort study which including a relatively small sample size. Against this, however, the differences observed were maintained in the main and subgroup analysis. Given it was a single-center study, the results may not be generalizable. They are, however, supported by other recent studies, and the suggestion that mesenteric inputs are potentially pathobiological is also borne out by observed outcomes after the Kono-S procedure (i.e., in which the mesentery is excluded from the anastomosis). It is noteworthy that rates of postoperative surgical recurrence after mesenteric resection or mesenteric exclusion appear similarly low (22,38). This study lacked an endoscopic arm and focused on postoperative outcomes and the requirement for reoperation. Neither of these is subject to the same level of variation associated with the interpretation of clinical and endoscopic findings (in general, patients do not subject themselves to unnecessary operations). Ideally, adding endoscopic and clinical recurrence would better in supporting our results. However, because of the retrospective nature of our study, it is difficult to collect postoperative endoscopic results 6 months or 1 year after surgery. The findings of this study prompt further investigation of the role of the mesentery in CD through randomized controlled trials. Several of these are now ongoing worldwide. If the potential benefits of mesenteric resection are borne out in these studies, then mesenteric resection may become a standard component in the surgical management of CD (61).

In summary, in patients undergoing surgery for CC, inclusion of the mesentery was associated with improved long-term outcomes relative to those in whom the mesentery was retained. Randomized controlled and multicenter trials are required to further investigate the position of the mesentery in the surgical management of CD.

## CONFLICTS OF INTERESTS

**Guarantor of the article:** Yi Li, MD, PhD.

**Specific author contributions:** Study concept and design: Y.Z., Y.L., and W.Z. Acquisition of data: Y.Z., W.Q., and L.H. Analysis or interpretation of data: Z.G., J.G., J.C.C., and B.S. Statistical analysis: Y.Z., Y.X., and L.C. Drafting of the manuscript: Y.Z., W.Q., L.H., and Y.X. Critical revision of the manuscript for important intellectual content: Y.L., W.Z., Z.hG., L.C., J.G., J.C.C., and B.S. Study supervision: Yi Li and Weiming Zhu. All authors agree the final approval of the version to be published.

**Financial support:** This work was partly supported by National Natural Science Foundation of China (Grant 81,670,471, 81,770,556, and 81,570,500) and Jiangsu Provincial Medical Youth Talent (QNRC2016900, to Yi Li).

**Potential competing interests:** None to report.

**Study Highlights**

**WHAT IS KNOWN**

✓ The mesentery is involved in Crohn's disease.
✓ Many factors were demonstrated to be associated with postoperative recurrence of Crohn's disease.

**WHAT IS NEW HERE**

✓ Extensive mesenteric excision in colorectal resection for Crohn's colitis is associated with reduced surgical recurrence.

## Supplementary Material

SUPPLEMENTARY MATERIAL
