# Supplementary material for: Role of Extended Mesenteric Excision in Postoperative Recurrence of Crohn's Colitis: A Single-Center Study
Source: Clin Transl Gastroenterol. 2021 Oct 1;12(10):e00407. doi: 10.14309/ctg.0000000000000407 (PMC8483874; doi:10.14309/ctg.0000000000000407)
Supplement: SUPPLEMENTARY MATERIAL [file ct9-12-e00407-s002.docx]

**Subgroup stoma creation analysis**

Twenty-five (19.8%) patients undergoing colorectal resection had diverting stomas, 14 (56%) in the LME group and 11 (44%) in the EME group. In the patients undergoing colorectal resection and stoma creation without following restoration, 5 (35.7%) patients in the LME group and 1 (9.1%) patient in the EME group developed postoperative surgical recurrence. The cumulative reoperation rates were 35.7% (n=14) during a mean 32.75$\pm$29.88 months and 9.1% (n=11) during 32.00 months in the LME and EME group (p=0.34), respectively **(Supplementary Figure A)**. Kaplan-Meier survival curves and log-rank test of the 2 groups were shown in **Supplementary Figure B**. There was a trend of reduced postoperative surgical recurrence in the EME group as compared with LME (p=0.21). No factors of postoperative surgical recurrence were found in this subgroup analysis (data were not shown). Multivariate analysis was not performed due to the small sample size in this subgroup.
